# Expansion of eigenvalues of the perturbed discrete bilaplacian

**DOI:** 10.1007/s00605-022-01678-1

**Published:** 2022-02-05

**Authors:** Shokhrukh Yu. Kholmatov, Ahmad Khalkhuzhaev, Mardon Pardabaev

**Affiliations:** 1 University of Vienna, Oskar-Morgenstern Platz 1, Vienna, 1090 Austria; 2 Samarkand State University, University boulevard 3, Samarkand, Uzbekistan 140104

**Keywords:** Discrete bilaplacian, Essential spectrum, Discrete spectrum, Eigenvalues, Asymptotics, Expansion, 47A10, 47A55, 47A75, 41A60

## Abstract

We consider the family

$$\begin{aligned} {\widehat{{ H}}}_\mu := {\widehat{\varDelta }} {\widehat{\varDelta }} - \mu {\widehat{{ V}}},\qquad \mu \in {\mathbb {R}}, \end{aligned}$$

of discrete Schrödinger-type operators in *d*-dimensional lattice ${\mathbb {Z}}^d$, where ${\widehat{\varDelta }}$ is the discrete Laplacian and ${\widehat{{ V}}}$ is of rank-one. We prove that there exist coupling constant thresholds $\mu _o,\mu ^o\ge 0$ such that for any $\mu \in [-\mu ^o,\mu _o]$ the discrete spectrum of ${\widehat{{ H}_\mu }}$ is empty and for any $\mu \in {\mathbb {R}}\setminus [-\mu ^o,\mu _o]$ the discrete spectrum of ${\widehat{{ H}_\mu }}$ is a singleton $\{e(\mu )\},$ and $e(\mu )<0$ for $\mu >\mu _o$ and $e(\mu )>4d^2$ for $\mu <-\mu ^o.$ Moreover, we study the asymptotics of $e(\mu )$ as $\mu \searrow \mu _o$ and $\mu \nearrow -\mu ^o$ as well as $\mu \rightarrow \pm \infty .$ The asymptotics highly depends on *d* and ${\widehat{{ V}}}.$

## Introduction

In this paper we investigate the spectral properties of the perturbed discrete biharmonic operator

1.1 $$\begin{aligned} {\widehat{{ H}}}_{{\mu }}:={\widehat{\varDelta }} {\widehat{\varDelta }} - \mu {\widehat{{ V}}},\qquad \mu \in {\mathbb {R}}, \end{aligned}$$

in the *d*-dimensional cubical lattice ${\mathbb {Z}}^d,$ where ${\widehat{\varDelta }}$ is the discrete Laplacian and ${\widehat{{ V}}}$ is a is rank-one potential with a generating potential ${\widehat{v}}.$ This model is associated to a one-particle system in ${\mathbb {Z}}^d$ with a potential field ${\widehat{v}},$ in which the particle freely “jumps” from a node *X* of the lattice not only to one of its nearest neighbors *Y* (similar to the discrete Laplacian case), but also to the nearest neighbors of the node *Y*. From the mathematical point of view, the discrete bilaplacian represents a discrete Schrödinger operator with a degenerate bottom, i.e., ${\widehat{\varDelta }}{\widehat{\varDelta }}$ is unitarily equivalent to a multiplication operator by a function ${\mathfrak {e}}$ which behaves as $o(|p-p_0|^2)$ close to its minimum point $p_0.$

The spectral properties of discrete Schrödinger operators with non-degenerate bottom (i.e., ${\mathfrak {e}}$ behaves as $O(|p-p_0|^2)$ close to its minimum point $p_0$), in particular with discrete Laplacian, have been extensively studied in recent years (see e.g. [1, 2, 7, 8, 10, 11, 20, 21, 23, 26, 28] and references therein) because of their applications in the theory of ultracold atoms in optical lattices [16, 24, 35, 36]. In particular, it is well-known that the existence of the discrete spectrum is strongly connected to the threshold phenomenon [18, 20–22], which plays an role in the existence the Efimov effect in three-body systems [31, 32, 34]: *if any two-body subsystem in a three-body system has no bound state below its essential spectrum and at least two two-body subsystem has a zero-energy resonance, then the corresponding three-body system has infinitely many bound states whose energies accumulate at the lower edge of the three-body essential spectrum*.

Recall that the Efimov effect may appear only for certain attractive systems of particles [29]. However, recent experimental results in the theory of ultracold atoms in an optical lattice have shown that two-particle systems can have repulsive bound states and resonances (see e.g. [36]), thus, one expects the Efimov effect to hold also for some repulsive three-particle systems in ${\mathbb {Z}}^3$.

The strict mathematical justification of the Effect effect including the asymptotics for the number of negative eigenvalues of the three-body Hamiltonian has been successfully established in 3-space dimensions (for both ${\mathbb {R}}^3$ and ${\mathbb {Z}}^3$) see e.g., [1, 4, 13, 19, 29, 31, 32, 34] and the references therein. In particular, the non-degeneracy of the bottom of the (reduced) one-particle Schrödinger operator played an important role in the study of resonance states of the associated two-body system [1, 31]. Another keypoint in the proof of the Efimov effect in ${\mathbb {Z}}^3$ was the asymptotics of the (unique) smallest eigenvalue of the (reduced) one-particle discrete Schrödinger operator which creates a singularity in the kernel of a Birman-Schwinger-type operator which used to obtain an asymptotics to the number of three-body bound states.

To the best of our knowledge, there are no published results related to the Efimov effect in lattice three-body systems in which associated (reduced) one-body Schrödinger operator has degenerate bottom.

We also recall that fourth order elliptic operators in ${\mathbb {R}}^d$ in particular, the biharmonic operator, play also a central role in a wide class of physical models such as linear elasticity theory, rigidity problems (for instance, construction of suspension bridges) and in streamfunction formulation of Stokes flows (see e.g. [9, 25, 27] and references therein). Moreover, recent investigations have shown that the Laplace and biharmonic operators have high potential in image compression with the optimized and sufficiently sparse stored data [15]. The need for corresponding numerical simulations has led to a vast literature devoted to a variety of discrete approximations to the solutions of fourth order equations [5, 12, 33]. The question of stability of such models is basically related to their spectral properties and therefore, numerous studies have been dedicated to the numerical evaluation of the eigenvalues [3, 6, 30].

The aim of the present paper is the study of the existence and asymptotics of eigenvalues as well as threshold resonance and bound states of ${\widehat{{ H}_\mu }}$ defined in (1.1), which corresponds to the one-body Schrödinger operator with degenerate bottom. Namely, we study the discrete spectrum of ${\widehat{{ H}_\mu }}$ depending on $\mu$ and on ${\widehat{v}}.$ For simplicity we assume the generator ${\widehat{v}}$ of ${\widehat{{ V}}}$ to decay exponentially at infinity, however, we urge that our methods can also be adjusted to less regular cases (see Remark 2.6). Since the spectrum of ${\widehat{\varDelta }}$ consists of [0, 2*d*] (see e.g., [1]), by the compactness of ${\widehat{{ V}}}$ and Weyl’s Theorem, the essential spectrum of ${\widehat{{ H}_\mu }}$ fills the segment $[0,4d^2]$ independently of $\mu .$ Moreover, the essential spectrum does not give birth to a new eigenvalue while $\mu$ runs in some real interval $[-\mu ^o,\mu _o],$ and it turns out as soon as $\mu$ leaves this interval through $\mu _o$ resp. through $-\mu ^o,$ a unique negative resp. a unique positive eigenvalue $e(\mu )$ releases from the essential spectrum (Theorem 2.2).

Now we are interested in the absorption rate of $e(\mu )$ as $\mu \rightarrow \mu _o$ and $\mu \rightarrow -\mu ^o.$ The associated asymptotics are highly dependent not only on the dimension *d* of the lattice (as in the discrete Laplacian case [20, 21]), but also values on the multiplicity $2n_o$ and $2n^o$ of $0\in \{v=0\}$ (if $v(0)=0$) and $\vec \pi \in \{v=0\}$ (if $v(\vec \pi )=0$), respectively. More precisely, depending on *d* and $n_o$, $e(\mu )$ has a convergent expansion

- in $$(\mu -\mu _o)^{1/3}$$ for $$2n_o+d=1,7;$$
- in $$\mu -\mu _o$$ for $$2n_o+d=3,5;$$
- in $(\mu -\mu _o)^{1/4}$ for $2n_o+d\ge 9$ with *d* odd;
- in $\mu - \mu _o$ and $-(\mu - \mu _o)\ln (\mu - \mu _o)$ for $2n_o+d=2,6;$
- in $\mu -\mu _o$ and $e^{-1/(\mu -\mu _o)}$ for $2n_o+d=4;$
- in $(\mu -\mu _o)^{1/2},$ $-(\mu -\mu _o)\ln (\mu -\mu _o),$ $(-\frac{1}{\ln (\mu -\mu _o)})^{1/2}$ and $-\frac{\ln \ln (\mu -\mu _o)^{-1}}{\ln (\mu -\mu _o)}$ for $2n_o+d=8;$
- in $(\mu -\mu _o)^{1/2}$ and $-(\mu -\mu _o)^{1/2}\ln (\mu -\mu _o)$ for $2n^o+d\ge 10$ with *d* even

(see Theorem 2.4). Moreover, resonance states of 0-energy, i.e. non-zero solutions *f* of ${\widehat{{ H}}}_{\mu _o}f=0$ not belonging to $\ell ^2({\mathbb {Z}}^d)$ appear if and only if $2n_o+d\in \{5,6,7,8\}.$ Recall that the emergence of 0-energy resonances in more lattice dimensions could allow the Efimov effect to be observed in other dimensions than $d=3.$

Furthermore, observing that the top ${\mathfrak {e}}(\vec \pi )=4d^2$ of the essential spectrum is non-degenerate, one expects the asymptotics of $e(\mu )$ as $\mu \rightarrow -\mu ^o$ to be similar as in the discrete Laplacian case [20, 21]; more precisely, depending on *d* and $n^o,$ $e(\mu )$ has a convergent expansion

- in $$\mu +\mu ^o$$ for $$2n^o+d=1,3;$$
- in $(\mu +\mu ^o)^{1/2}$ for $2n^o+d\ge 5$ with *d* odd;
- in $\mu +\mu ^o$ and $e^{-1/(\mu +\mu ^o)}$ for $2n_o+d=2;$
- in $\mu +\mu ^o,$ $-\frac{1}{\ln (\mu +\mu ^o)}$ and $-\frac{\ln \ln (\mu +\mu ^o)^{-1}}{\ln (\mu +\mu ^o)}$ for $2n_o+d=4;$
- in $\mu +\mu ^o$ and $-(\mu +\mu ^o)\ln (\mu +\mu ^o)$ for $2n^o+d\ge 6$ with *d* even

(see Theorem 2.5). Moreover, the resonance states of energy $4d^2$, i.e. non-zero solutions *f* of ${\widehat{{ H}}}_{-\mu ^o}f=4d^2f$ not belonging to $\ell ^2({\mathbb {Z}}^d)$ appear if and only if $2n_o+d=3,4.$

The threshold analysis for more general class of nonlocal discrete Schrödinger operators with $\delta$-potential of type

$$\begin{aligned} {\widehat{H}}_\mu = \Psi (-{\widehat{\varDelta }}) + \mu \delta _{x0}, \end{aligned}$$

can be found in [14], where $\Psi$ is some strictly increasing $C^1$-function and $\delta _{x0}$ is the Dirac’s delta-function supported at 0. Besides the existence of eigenvalues, authors of [14] classify (embedded) threshold resonances and threshold eigenvalues depending on the behaviour of $\Psi$ at the edges of the essential spectrum of $-{\widehat{\varDelta }}$ and on the lattice dimension *d*. The eigenvalue expansions for the discrete bilaplacian with $\delta$-perturbation have been established in [17] for $d=1$ using the complex analytic methods.

The paper is organized as follows. In Sect. 2 after introducing some preliminaries we state the main results of the paper. In Theorem 2.2 we establish necessary and sufficient conditions for non-emptiness of the discrete spectrum of ${\widehat{{ H}_\mu }},$ and in case of existence, we study the location and the uniqueness, analiticity, monotonicity and convexity properties of eigenvalues $e(\mu )$ as a function of $\mu .$ In particular, we study the asymptotics of $e(\mu )$ as $\mu \rightarrow \mu _o$ and $\mu \rightarrow -\mu ^o$ as well as $\mu \rightarrow \pm \infty .$ As discussed above in Theorems 2.4 and 2.5 we obtain expansions of $e(\mu )$ for small and positive $\mu -\mu _o$ and $\mu +\mu ^o$. In Sect. 3 we prove the main results. The main idea of the proof is to obtain a nonlinear equation $\Delta (\mu ;z)=0$ with respect to the eigenvalue $z=e(\mu )$ of ${\widehat{{ H}_\mu }}$ and then study properties of $\Delta (\mu ;z).$ Finally, in appendix Section A we obtain the asymptotics of certain integrals related to $\Delta (\mu ;z)$ which will be used in the proofs of main results.

### Data availability statement

We confirm that the current manuscript has no associated data.

## Preliminary and main results

Let ${\mathbb {Z}}^d$ be the *d*-dimensional lattice and $\ell ^2({\mathbb {Z}}^d)$ be the Hilbert space of square-summable functions on ${\mathbb {Z}}^d.$ Consider the family

$$\begin{aligned} {\widehat{{ H}_\mu }}:={\widehat{{ H}}}_0 - \mu {\widehat{{ V}}},\qquad \mu \ge 0, \end{aligned}$$

of self-adjoint bounded discrete Schrödinger operators in $\ell ^2({\mathbb {Z}}^d).$ Here ${\widehat{{ H}}}_0:={\widehat{\varDelta }}{\widehat{\varDelta }}$ is discrete bilaplacian, where

$$\begin{aligned} {\widehat{\varDelta }} f(x) = \frac{1}{2}\,\sum \limits _{|s|=1} [f(x) - f(x+s)],\qquad f\in \ell ^2({\mathbb {Z}}^d), \end{aligned}$$

is the discrete Laplacian, and ${\widehat{{ V}}}$ is a rank-one operator

$$\begin{aligned} {\widehat{{ V}}} {\widehat{f}}(x) = {\widehat{v}}(x) \sum \limits _{y\in {\mathbb {Z}}^d} {{\widehat{v}}(y)} {\widehat{f}}(y), \end{aligned}$$

where ${\widehat{v}}\in \ell ^2({\mathbb {Z}}^d)\setminus \{0\}$ is a given real-valued function.

Let ${\mathbb {T}}^d$ be the *d*-dimensional torus equipped with the Haar measure and $L^2({\mathbb {T}}^d)$ be the Hilbert space of square-integrable functions on ${\mathbb {T}}^d.$ By ${\mathcal {F}}$ we denote the the standard Fourier transform

$$\begin{aligned} {\mathcal {F}}:\ell ^2({\mathbb {Z}}^d)\rightarrow L^2({\mathbb {T}}^d),\qquad {\mathcal {F}}{\widehat{f}}(p) = \frac{1}{(2\pi )^{d/2}}\,\sum \limits _{x\in {\mathbb {Z}}^d} {\widehat{f}}(x) e^{ixp}. \end{aligned}$$

Further we always assume that ${\widehat{v}}$ and its Fourier image

$$\begin{aligned} v(p):={\mathcal {F}}{\widehat{v}}(p)=\frac{1}{(2\pi )^{d/2}}\sum \limits _{x\in {\mathbb {Z}}^d} {\widehat{v}}(x)e^{ix\cdot p} \end{aligned}$$

satisfy the following assumptions: 
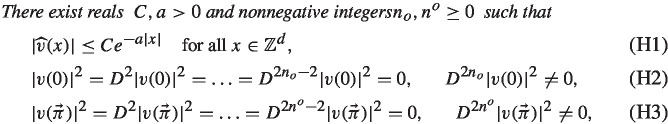
 here $D^jf(p)$ is the *j*-th order differential of *f* at *p*,  i.e. the *j*-th order symmetric tensor

$$\begin{aligned} D^jf(p)[\underbrace{w,\ldots ,w}_{j-times}]= & {} \sum \limits _{i_1+\ldots +i_d =j,i_k\ge 0} \frac{\partial ^jf(p)}{\partial ^{i_1} p_1\ldots \partial ^{i_d}p_d} \,w_1^{i_1}\ldots w_d^{i_d},\nonumber \\&\qquad \quad w=(w_1,\ldots ,w_d)\in {\mathbb {R}}^d, \end{aligned}$$

and $\vec {\pi }=(\pi ,\ldots ,\pi )\in {\mathbb {T}}^d.$ Notice that under assumption (H1), *v* is analytic on ${\mathbb {T}}^d.$

Recall that $\sigma ({\widehat{\varDelta }}) = \sigma _{\mathrm {ess}}({\widehat{\varDelta }}) =[0,2d]$ (see e.g. [1]). Hence, $\sigma ({\widehat{{ H}}}_0) = \sigma _{\mathrm {ess}}({\widehat{{ H}}}_0) =[0,4d^2],$ and by the compactness of ${\widehat{{ V}}}$ and Weyl’s Theorem,

$$\begin{aligned} \sigma _{\mathrm {ess}}({\widehat{{ H}_\mu }}) = \sigma _{\mathrm {ess}}({\widehat{{ H}}}_0) =[0,4d^2] \end{aligned}$$

for any $$\mu \in {\mathbb {R}}.$$

Before stating the main results let us introduce the constants

2.2 $$\begin{aligned}&\mu _o:= \Big (\int _{{\mathbb {T}}^d} \frac{|v(q)|^2dq}{{\mathfrak {e}}(q)}\Big )^{-1},\qquad \mu ^o:= \Big (\int _{{\mathbb {T}}^d} \frac{|v(q)|^2dq}{4d^2 - {\mathfrak {e}}(q)}\Big )^{-1}, \end{aligned}$$

2.3 $$\begin{aligned}&{\widehat{c}}_v:= \int _{{\mathbb {T}}^d} \frac{|v(q)|^2dq}{{\mathfrak {e}}(q)^2},\qquad {\widehat{C}}_v:= \int _{{\mathbb {T}}^d} \frac{|v(q)|^2dq}{(4d^2 - {\mathfrak {e}}(q))^2}, \end{aligned}$$

and

2.4 $$\begin{aligned} c_v:=&\frac{2^{2n_o+d}}{(2n_o)!}\, \int _{{\mathbb {S}}^{d-1}} D^{2n_o}|v(0)|^2[w,\ldots , w]\,d{\mathcal {H}}^{d-1}(w), \end{aligned}$$

2.5 $$\begin{aligned} C_v:=&\frac{2^{2n^o+d-1}}{(8d)^{n^o+d/2}\,(2n^o)!}\, \int _{{\mathbb {S}}^{d-1}} D^{2n^o}|v(\vec \pi )|^2[w,\ldots ,w]\,d{\mathcal {H}}^{d-1}(w), \end{aligned}$$

where ${\mathbb {S}}^{d-1}$ is the unit sphere in ${\mathbb {R}}^d$ and

$$\begin{aligned} {\mathfrak {e}}(q): =\left( \sum \limits _{i=1}^d ( 1 - \cos q_i)\right) ^2. \end{aligned}$$

### Remark 2.1

Under assumptions (H1)–(H3), $\mu _o,\mu ^o\ge 0,$ $c_v,C_v>0,$ and ${\widehat{c}}_v,{\widehat{C}}_v\in (0,+\infty ].$ Moreover, by Propositions A.1 and A.2:

- $\mu _o=0$ (resp. $\mu ^o=0$) if and only if $2n_o+d\le 4$ (resp. $2n^o+d\le 2$);
- ${\widehat{c}}_v<\infty$ (resp. ${\widehat{C}}_v<\infty$) if $2n_o+d\ge 9$ (resp. $2n^o+d\ge 5$).

### Main results

First we concern with the existence of the discrete spectrum of ${\widehat{{ H}_\mu }}.$

#### Theorem 2.2

Let $\mu _o,\mu ^o\ge 0$ be given by (2.2). Then $\sigma _{\mathrm {disc}}({\widehat{{ H}_\mu }})=\emptyset$ for any $\mu \in [-\mu ^o,\mu _o]$ and $\sigma _{\mathrm {disc}}({\widehat{{ H}_\mu }})$ is a singleton $\{e(\mu )\}$ for any $\mu \in {\mathbb {R}}\setminus [-\mu ^o,\mu _o].$ Moreover, the associated eigenfunction ${\widehat{f}}_\mu$ to $e(\mu )$ is given by ${\widehat{f}}_\mu :={\mathcal {F}}^*f_\mu ,$ where

$$\begin{aligned} f_\mu (p) = \frac{v(p)}{{\mathfrak {e}}(p) - e(\mu )}. \end{aligned}$$

Furthermore, if $\mu <-\mu ^o$ (resp. $\mu >\mu _o$), then $e(\mu )>4d^2$ (resp. $e(\mu )<0$). Moreover, the function $\mu \in {\mathbb {R}}\setminus [-\mu ^o,\mu _o]\mapsto e(\mu )$ is real-analytic strictly decreasing, convex in $(-\infty ,-\mu ^o)$ and concave in $(\mu _o,+\infty ),$ and satisfies

2.6 $$\begin{aligned} \lim \limits _{\mu \searrow \mu _o} e(\mu ) =0\qquad \text {and}\qquad \lim \limits _{\mu \nearrow -\mu ^o} e(\mu ) =4d^2 \end{aligned}$$

and

2.7 $$\begin{aligned} \lim \limits _{\mu \rightarrow \pm \infty } \frac{e(\mu )}{\mu } = - \int _{{\mathbb {T}}^d}|v(q)|^2dq. \end{aligned}$$

Next we study the threshold resonances of ${\widehat{{ H}_\mu }}.$

#### Theorem 2.3

Let $n_o,n^o\ge 0$ be given by (H2)–(H3).

- Let $2n_o+d\ge 5.$ Then ${\widehat{f}}:={\mathcal {F}}^*f\in c_0({\mathbb {Z}}^d),$ i.e., ${\widehat{f}}(x)\rightarrow 0$ as $|x|\rightarrow +\infty ,$ where
  $$\begin{aligned} f(p) = \frac{v(p)}{{\mathfrak {e}}(p)} \in L^1({\mathbb {T}}^d). \end{aligned}$$
  Moreover, ${\widehat{f}}\in c_0({\mathbb {Z}}^d)\setminus \ell ^2({\mathbb {Z}}^d)$ for $2n_o+d\in \{5,6,7,8\},$ ${\widehat{f}}\in \ell ^2({\mathbb {Z}}^d)$ for $2n_o+d\ge 9,$ and ${\widehat{f}}$ solves the equation ${\widehat{{ H}}}_{\mu _o} f=0.$
- Let $2n^o+d\ge 3.$ Then ${\widehat{g}}:={\mathcal {F}}^*g\in \ell ^0({\mathbb {Z}}^d),$ where
  $$\begin{aligned} g(p) = \frac{v(p)}{4d^2 - {\mathfrak {e}}(p)}. \end{aligned}$$
  Moreover, ${\widehat{g}}\in \ell ^0({\mathbb {Z}}^d)\setminus \ell ^2({\mathbb {Z}}^d)$ for $2n^o+d\in \{3,4\},$ ${\widehat{g}}\in \ell ^2({\mathbb {Z}}^d)$ for $2n^o+d\ge 5,$ and ${\widehat{g}}$ solves the equation ${\widehat{{ H}}}_{-\mu ^o} f=4d^2f.$

We recall that in the literature the non-zero solutions of equations ${\widehat{{ H}_\mu }} {\widehat{f}}=0$ and ${\widehat{{ H}_\mu }}{\widehat{g}}= 4d^2 {\widehat{g}}$ not belonging to $\ell ^2({\mathbb {Z}}^d)$ are called the *resonance states* [1, 2].

Now we study the rate of the convergences in (2.6).

#### Theorem 2.4

(Expansions of $e(\mu )$ at $\mu =\mu _o$) For $\mu >\mu _o$ let $e(\mu )<0$ be the eigenvalue of ${\widehat{{ H}_\mu }}.$

- Suppose that *d* is odd:
  1. if $2n_o+d=1,3,$ then $\mu _o=0$ and for sufficiently small and positive $\mu ,$
    $$\begin{aligned} (- e(\mu ))^{1/4}= {\left\{ \begin{array}{ll} \Big (\frac{\pi c_v}{4}\Big )^{1/3}\,\mu ^{1/3} + \sum \limits _{n\ge 1} c_{1,n} \mu ^{\frac{n+1}{3}}, &{} 2n_o+d=1,\\ \frac{\pi c_v}{8}\,\mu + \sum \limits _{n\ge 1} c_{3,n} \mu ^{n+1}, &{} 2n_o+d=3, \end{array}\right. } \end{aligned}$$
    where $\{c_{1,n}\}$ and $\{c_{3,n}\}$ are some real coefficients;
  2. if $2n_o+d=5,7,$ then $\mu _o>0$ and for sufficiently small and positive $\mu -\mu _o,$
    $$\begin{aligned}&(- e(\mu ))^{1/4}\\&\quad = {\left\{ \begin{array}{ll} \frac{8}{\pi c_v\mu _o^2} \, (\mu -\mu _o) +\sum \limits _{n\ge 1} c_{5,n} (\mu -\mu _o)^{n+1}, &{} 2n_o+d=5,\\ \Big (\frac{8}{\pi c_v\mu _o^2}\Big )^{1/3}\, (\mu -\mu _o)^{1/3} + \sum \limits _{n\ge 1} c_{7,n} (\mu -\mu _o)^{\frac{n+1}{3}}, &{} 2n_o+d=7, \end{array}\right. } \end{aligned}$$
    where $\{c_{5,n}\}$ and $\{c_{7,n}\}$ are some real coefficients;
  3. if $2n_o+d\ge 9,$ then $\mu _o>0$ and for sufficiently small and positive $\mu -\mu _o,$
    $$\begin{aligned} (- e(\mu ))^{1/4}= (\mu _o^2{\widehat{c}}_v)^{-1/4}\,(\mu -\mu _o)^{1/4} + \sum \limits _{n\ge 1} c_{9,n} (\mu -\mu _o)^{n/4}, \end{aligned}$$
    where $\{c_{9,n}\}$ are some real coefficients.
- Suppose that *d* is even:
  1. if $2n_o+d=2,4,$ then $\mu _o=0$ and for sufficiently small and positive $\mu ,$
    $$\begin{aligned}&(- e(\mu ))^{1/2}\\&\quad = {\left\{ \begin{array}{ll} \frac{\pi c_v}{8}\,\mu + \sum \limits _{n+m\ge 1,n,m\ge 0} c_{2,nm} \mu ^{n+1}(-\mu \ln \mu )^{m}, &{} 2n_o+d=2,\\ ce^{-\frac{8}{c_v\mu }} +\sum \limits _{n+m\ge 1,n,m\ge 0} c_{4,nm} \mu ^{n+1}\left( \frac{1}{\mu }\,e^{-\frac{8}{c_v\mu }}\right) ^{m+1}, &{} 2n_o+d=4, \end{array}\right. } \end{aligned}$$
    where $\{c_{2,nm}\}$ and $\{c_{4,nm}\}$ are some real coefficients and $c>0;$
  2. if $2n_o+d=6,8,$ then $\mu _o>0$ and for sufficiently small and positive $\mu -\mu _o,$
    $$\begin{aligned}&(- e(\mu ))^{1/2}\\&\quad = {\left\{ \begin{array}{ll} \frac{8}{\pi c_v\mu _o^2}\,\tau ^2 + \sum \limits _{n+m\ge 1,n,m\ge 0} c_{6,nm} \tau ^{2n+2}\theta ^m, &{} 2n_o+d=6,\\ \Big (\frac{8}{c_v\mu _o^2}\Big )^{1/2}\,\tau \sigma + \sum \limits _{n+m+k\ge 1,n,m,k\ge 0} c_{8,nmk} \tau ^{n+1} \sigma ^{m+1}\eta ^k, &{} 2n_o+d=8, \end{array}\right. } \end{aligned}$$
    where $\{c_{4,nm}\}$ and $\{c_{8,nmk}\}$ are some real coefficients and
    2.8 $$\begin{aligned} \tau :=(\mu -\mu _o)^{1/2},\,\,\, \theta :=-\tau ^2\ln \tau ,\,\,\, \sigma :=\Big (-\frac{1}{\ln \tau }\Big )^{1/2},\,\,\, \eta :=-\frac{\ln \ln \tau ^{-1}}{\ln \tau },\nonumber \\ \end{aligned}$$
  3. if $2n_o+d\ge 10,$ then $\mu _o>0$ and for sufficiently small and positive $\mu -\mu _o,$
    $$\begin{aligned} (- e(\mu ))^{1/2}= (\mu _o^2{\widehat{c}}_v)^{-1/2}\,\tau + \sum \limits _{n+m\ge 1,n,m\ge 0} c_{10,nm} \tau ^{n+1}\theta ^m, \end{aligned}$$
    where $\{c_{10,nm}\}$ are some real coefficients.

Here $c_v>0$ and ${\widehat{c}}_v>0$ are given by (2.4) and (2.3), respectively.

#### Theorem 2.5

(**Expansions of** $e(\mu )$ **at** $\mu =-\mu ^o$) For let $\mu <-\mu ^o$ let $e(\mu )>4d^2$ be the eigenvalue of ${\widehat{{ H}_\mu }}.$

- Suppose that *d* is odd:
  1. if $2n^o+d=1,$ then $\mu ^o=0$ and for sufficiently small and negative $\mu ,$
    $$\begin{aligned} (e(\mu ) - 4d^2)^{1/2}= -\pi C_v\,\mu + \sum \limits _{n\ge 1} C_{1,n} \mu ^{n+1}, \end{aligned}$$
    where $\{C_{1,n}\}$ are some real coefficients;
  2. if $2n^o+d=3,$ then $\mu ^o>0$ and for sufficiently small and positive $\mu +\mu ^o,$
    $$\begin{aligned} (e(\mu ) - 4d^2)^{1/2}= (\pi C_v{\mu ^o}^2)^{-1}\,(\mu +\mu ^o) +\sum \limits _{n\ge 1} C_{3,n} (\mu +\mu ^o)^{n+1}, \end{aligned}$$
    where $\{C_{3,n}\}$ and $\{C_{7,n}\}$ are some real coefficients;
  3. if $2n^o+d\ge 5,$ then $\mu ^o>0$ and for sufficiently small and positive $\mu +\mu ^o,$
    $$\begin{aligned} (e(\mu ) - 4d^2)^{1/2}= ({\widehat{C}}_v {\mu ^o}^2)^{-1/2}\,(\mu +\mu ^o)^{1/2} + \sum \limits _{n\ge 1} C_{5,n} (\mu +\mu ^o)^{(n+1)/2}, \end{aligned}$$
    where $\{C_{5,n}\}$ are some real coefficients.
- Suppose that *d* is even:
  1. if $2n_o+d=2,$ then $\mu _o=0$ and for sufficiently small and negative $\mu ,$
    $$\begin{aligned} e(\mu ) - 4d^2= C\,e^{\frac{1}{C_v\mu }} +\sum \limits _{n+m\ge 1,n,m\ge 0} C_{2,nm} \mu ^{n+1}\left( -\frac{1}{\mu }\,e^{\frac{1}{C_v\mu }}\right) ^{m+1}, \end{aligned}$$
    where $\{C_{2,nm}\}$ are some real coefficients and $C>0;$
  2. if $2n_o+d=4,$ then $\mu ^o>0$ and for sufficiently small and positive $\mu +\mu ^o,$
    $$\begin{aligned} e(\mu ) - 4d^2= (C_v{\mu ^o}^2)^{-1}\,\mu \sigma + \sum \limits _{n+m+k\ge 1,n,m,k\ge 0} C_{4,nmk} \tau ^{n+1} \sigma ^{m+1}\eta ^k, \end{aligned}$$
    where $\{C_{4,nm}\}$ are some real coefficients and
    $$\begin{aligned} \tau :=\mu +\mu ^o,\qquad \sigma :=-\frac{1}{\ln \tau },\qquad \eta :=-\frac{\ln \ln \tau ^{-1}}{\ln \tau }; \end{aligned}$$
  3. if $2n_o+d\ge 6,$ then $\mu ^o>0$ and for sufficiently small and positive $\mu +\mu ^o,$
    $$\begin{aligned} e(\mu )-4d^2= & {} ({\widehat{C}}_v{\mu ^o}^2)^{-1}\,(\mu +\mu ^o)\\&+ \sum \limits _{n+m\ge 1,n,m\ge 0} C_{6,nm} (\mu +\mu ^o)^{n+1}[-(\mu +\mu ^o)\ln (\mu +\mu ^o)]^m, \end{aligned}$$
    where $\{C_{6,nm}\}$ are some real coefficients.

Here $C_v$ and ${\widehat{C}}_v$ are given by (2.5) and (2.3), respectively.

#### Remark 2.6

Few comments on the main results are in order.

1. The assertions of Theorem 2.2 hold in fact for any ${\widehat{v}}\in \ell ^2({\mathbb {Z}}^d)$ (see Remark 3.2);
2. Similar expansions of $e(\mu )$ in Theorems 2.4 and 2.5 at $\mu =\mu _o$ and $\mu =-\mu ^o,$ respectively, still hold for any exponentially decaying ${\widehat{v}}:{\mathbb {Z}}^d\rightarrow {\mathbb {C}}$ (see Remark 3.3);
3. If ${\widehat{v}}$ decays at most polynomially at infinity, i.e. ${\widehat{v}}(x) = O(|x|^{-\alpha })$ for some $\alpha >0,$ then instead of the expansions in Theorem 2.4 and 2.5 we obtain only asymptotics of $e(\mu )$ (see Remark 3.4).

## Proof of main results

In this section we prove the main results. By the Birman-Schwinger principle and the Fredholm Theorem we have

### Lemma 3.1

A complex number $z\in {\mathbb {C}}\setminus [0,4d^2]$ is an eigenvalue of ${\widehat{{ H}_\mu }}$ if and only if

$$\begin{aligned} \Delta (\mu ;z):=1 - \mu \int _{{\mathbb {T}}^d} \frac{|v(q)|^2dq}{{\mathfrak {e}}(q) - z}=0. \end{aligned}$$

### Proof of Theorem 2.2

By the definition of $\mu _o,$ for any $\mu <-\mu ^o:$

$$\begin{aligned} \lim \limits _{z\nearrow -\mu ^o} \Delta (\mu ;z) = 1 + \frac{\mu }{\mu ^o}<0,\qquad \lim \limits _{z\rightarrow +\infty } \Delta (\mu ;z) =1. \end{aligned}$$

Since $\Delta (\mu ;z)>1$ for $z<0$ and $\mu >-\mu ^o,$ in view of the strict monotonicity $\Delta (\mu ;\cdot )$ in $(4d^2,\infty ),$ for any $\mu <-\mu ^o$ there exists a unique $e(\mu )\in (4d^2,+\infty )$ such that $\Delta (\mu ;e(\mu ))=0.$ Analogously, for any $\mu >\mu _o$ there exists a unique $e(\mu )\in (-\infty ,0)$ such that $\Delta (\mu ;e(\mu ))=0.$ By the Implicit Function Theorem the function $\mu \in {\mathbb {R}}\setminus [-\mu ^o,\mu _o]\mapsto e(\mu )$ is real-analytic. Moreover, computing the derivatives of the implicit function $e(\mu )$ we find:

3.1 $$\begin{aligned} e'(\mu ) = - \frac{1}{\mu }\,\int _{{\mathbb {T}}^d} \frac{|v(q)|^2dq}{{\mathfrak {e}}(q) - e(\mu )}\,\Big ( \int _{{\mathbb {T}}^d} \frac{|v(q)|^2dq}{({\mathfrak {e}}(q) - e(\mu ))^2}\Big )^{-1},\qquad \mu \ne 0, \end{aligned}$$

thus, using $\mu ({\mathfrak {e}}(q) - e(\mu ))>0$ we get $e'(\mu )<0,$ i.e. $e(\cdot )$ is strictly decreasing in ${\mathbb {R}}\setminus \{0\}.$ Differentiating (3.1) one more time we get

$$\begin{aligned} e''(\mu ) = \frac{2e'(\mu )}{\mu }\,\left( 1-\mu e'(\mu )\,\int _{{\mathbb {T}}^d} \frac{|v(q)|^2dq}{({\mathfrak {e}}(q) - e(\mu ))^3}\,\left( \int _{{\mathbb {T}}^d} \frac{|v(q)|^2dq}{({\mathfrak {e}}(q) - e(\mu ))^2}\right) ^{-1}\right) . \end{aligned}$$

Therefore, $e''(\mu )>0$ (i.e. $e(\cdot )$ is strictly convex) for $\mu <0$ and $e''(\mu )<0$ (i.e. $e(\cdot )$ is strictly concave) for $\mu >0.$

To prove (2.7), first we let $\mu \rightarrow \pm \infty$ in

3.2 $$\begin{aligned} 1= \mu \int _{{\mathbb {T}}^d} \frac{|v(q)|^2dq}{{\mathfrak {e}}(q) - e(\mu )} \end{aligned}$$

and find $\lim \limits _{\mu \rightarrow \pm \infty } e(\mu )=\mp \infty .$ In particular, if $|\mu |$ is sufficiently large, $|\frac{{\mathfrak {e}}(q)}{e(\mu )}|<\frac{1}{2}$ and hence, by (3.2) and the Dominated Convergence Theorem,

$$\begin{aligned} \lim \limits _{\mu \rightarrow \pm \infty } \frac{e(\mu )}{\mu } = - \lim \limits _{\mu \rightarrow \pm \infty } \int _{{\mathbb {T}}^d}\frac{|v(q)|^2dq}{1- \frac{{\mathfrak {e}}(q)}{e(\mu )}} = -\int _{{\mathbb {T}}^d} |v(q)|^2dq. \end{aligned}$$

To prove that ${\widehat{f}}_\mu$ solves ${\widehat{{ H}_\mu }} {\widehat{f}}_\mu =e(\mu ){\widehat{f}}_\mu$ we consider the equivalent equality ${\mathcal {F}}{\widehat{{ H}_\mu }} {\mathcal {F}}^* f_\mu =e(\mu )f_\mu ,$ which is easily reduced to the equality $\Delta (\mu ;e(\mu ))=0.$ $\square$

### Remark 3.2

In view of Lemma 3.1 and the proof of Theorem 2.2, their assertions still hold for any $v\in \ell ^2({\mathbb {Z}}^d).$

### Proof of Theorem 2.3

We prove only (a), the proof of (b) being similar. Repeating the proof of the continuity (resp. differentiability) of $\mathfrak {l}_f$ at $z=0$ in Proposition A.1 one can show that $f\in L^1({\mathbb {T}}^d)\setminus L^2({\mathbb {T}}^d)$ for $2n_o+d\in \{5,6,7,8\}$ and $f\in L^2({\mathbb {T}}^d)$ for $2n_o+d\ge 9.$ Thus, by the Riemann-Lebesgue Lemma, ${\widehat{f}}\in \ell ^0({\mathbb {Z}}^d).$ To show that ${\widehat{{ H}}}_{\mu _o}{\widehat{f}}=0$ it suffices to observe that ${\mathcal {F}}{\widehat{{ H}}}_{\mu _0} {\mathcal {F}}^*f=0.$ $\square$

### Proof of Theorem 2.4

Since

3.3 $$\begin{aligned} |v(p)|^2= (2\pi )^{-d}\left( \sum \limits _{x\in {\mathbb {Z}}^d} {\widehat{v}}(x)\cos p\cdot x\right) ^2+(2\pi )^{-d}\left( \sum \limits _{x\in {\mathbb {Z}}^d} {\widehat{v}}(x)\sin p\cdot x\right) ^2,\nonumber \\ \end{aligned}$$

the function $p\in {\mathbb {T}}^d\mapsto |v(p)|^2$ is nonnegative even real-analytic function. Notice also that if $n_o\ge 1,$ then by the nonnegativity of $|v|^2,$ $p=0$ is a global minimum for $|v|^2.$ Therefore, the tensor $D^{2n_o}|v(0)|^2$ is positively definite and

$$\begin{aligned} c_v:=\frac{2^{2n_o+d}}{(2n_o)!} \int _{{\mathbb {S}}^{d-1}} D^{2n_o} |v(0)|^2[w,\ldots ,w]d{\mathcal {H}}^{d-1}>0. \end{aligned}$$

Note that

$$\begin{aligned} {\widehat{c}}_v = \mathfrak {l}_{|v|^2}'(0)= \int _{{\mathbb {T}}^d}\frac{|v(q)|^2dq}{{\mathfrak {e}}(q)^2}, \end{aligned}$$

where $\mathfrak {l}_f$ is defined in (A.1). By Proposition A.1, $f(p)=\frac{v(p)}{{\mathfrak {e}}(p)}\in L^2({\mathbb {T}}^d)$ if and only if $2n_o+d\ge 9.$ Moreover, by definition, $\mu _o>0$ and $\Delta (\mu _o;0)=0$ for $2n_o+d\ge 5,$ and hence, as in the proof of Lemma 3.1 for such *d* one can show that ${ H}_{\mu _o}f=0.$

In view of the strict monotonicity and (2.6) there exists a unique $\mu _1>0$ such that $e(\mu )\in (-\frac{1}{128},0)$ for any $\mu \in (0,\mu _1).$ Since

3.4 $$\begin{aligned} \mu = (\mathfrak {l}_{|v|^2}(e(\mu )))^{-1}, \end{aligned}$$

we can use Proposition A.1 with $f=|v|^2$ and $e:=e(\mu ),$ to find the expansions of the inverse function $\mu :=\mu (e).$ Then applying the appropriate versions of the Implicit Function Theorem in analytical case we get the expansions of $e=e(\mu ).$ Notice that from (A.3) and (A.4) as well as (3.5) it follows that $\mu _o=0$ for $2n_o+d \le 4$ and $\mu _o=\Big (\int _{{\mathbb {T}}^d} \frac{|v(q)|^2dq}{{\mathfrak {e}}(q)}\Big )^{-1}>0$ for $2n_o+d\ge 5.$

(a) Suppose that *d* is odd. In view of the expansions (A.3) of $\mathfrak {l}_f$, in this case, (3.4) is reduced to the inverting the equation

3.5 $$\begin{aligned} \mu = g(\alpha ), \end{aligned}$$

where $\alpha :=(-e)^{1/4}$ and *g* is an analytic function around $\alpha =0.$

*Case* $2n_o+d=1.$ In this case by (A.3),

$$\begin{aligned} g(\alpha ) := \frac{\alpha ^3}{c_1^3 + \sum \nolimits _{n\ge 1} a_n \alpha ^n}, \end{aligned}$$

where $\{a_n \}\subset {\mathbb {R}}$ and $c_1:=(\pi c_v/4)^{1/3}$ and (3.5) is equivalently represented as

3.6 $$\begin{aligned} \alpha = \mu \left( c_1^3 + \sum \limits _{n\ge 1} a_n \alpha ^{n}\right) ^{1/3}, \end{aligned}$$

where $\mu =\mu ^{1/3}.$ Now setting

3.7 $$\begin{aligned} \alpha = \mu (c_1 + u), \end{aligned}$$

and using the Taylor series of $(c_1^3+x)^{1/3},$ for $\mu$ and *u* sufficiently small we rewrite (3.6) as

3.8 $$\begin{aligned} F(u,\mu ):= u - \sum \limits _{n\ge 1} {\tilde{a}}_n \mu ^n(c_1 + u)^n =0, \end{aligned}$$

where $F(\cdot ,\cdot )$ is analytic at $(u,\mu )=(0,0),$ $F(0,0)=0$ and $F_u(0,0) =1.$ Hence, by the Implicit Function Theorem, there exists $\gamma _1>0$ such that for $|\mu |<\gamma _1,$ (3.8) has a unique real-analytic solution $u=u(\mu )$ which can be represented as an absolutely convergent series $u=\sum \nolimits _{n\ge 1} b_n\mu ^n.$ Putting this in (3.7) and recalling the definitions of $\alpha$ and $\mu$ we get the expansion of $(-e(\mu ))^{1/4}$ for $\mu >0$ small.

*Case* $$2n_o+d=3.$$ By (A.3),

3.9 $$\begin{aligned} g(\alpha )=\alpha \left( c_3 + \sum \limits _{n\ge 1} a_n \alpha ^n\right) ^{-1}, \end{aligned}$$

where $\{a_n\}\subset {\mathbb {R}}$ and $c_3:=\pi c_v/8,$ and hence, (3.5) is represented as

$$\begin{aligned} \alpha = \mu \left( c_3 + \sum \limits _{n\ge 1} a_n \alpha ^n\right) . \end{aligned}$$

Then setting $\alpha =\mu (c_3+u)$ we rewrite (3.9) in the form (3.8), and as in the case of $2n_o+d=1,$ we get the expansion of $(-e(\mu ))^{1/4}.$

*Case* $2n_o+d=5.$ In this case by (A.3)

$$\begin{aligned} g(\alpha )= \left( \frac{1}{\mu _o} - \frac{\pi c_v\alpha }{8}\left( 1+ \sum \limits _{n\ge 1}a_n\alpha ^n \right) \right) ^{-1}, \end{aligned}$$

where $\{a_n\}\subset {\mathbb {R}},$ and hence, by (3.5),

3.10 $$\begin{aligned} \frac{\mu - \mu _o}{\mu \mu _o}= \frac{\pi c_v\alpha }{8}\left( 1+ \sum \limits _{n\ge 1}a_n\alpha ^n \right) . \end{aligned}$$

Note that if $|\mu -\mu _o|<\mu _o,$ then

3.11 $$\begin{aligned} \frac{\mu - \mu _o}{\mu \mu _o} = \frac{\mu - \mu _o}{{\mu _o}^2+ \mu _o(\mu - \mu _o)} = \frac{\mu - \mu _o}{{\mu _o}^2} \sum \limits _{n\ge 0} \left( \frac{\mu - \mu _o}{\mu _o}\right) ^n, \end{aligned}$$

thus from (3.10) we get

$$\begin{aligned} \alpha = (\mu - \mu _o)\left( c_5 + c_5 \sum \limits _{n\ge 1} \mu _o^{-n} (\mu - \mu _o)^n\right) \,\left( 1+ \sum \limits _{n\ge 1}a_n\alpha ^n \right) ^{-1}. \end{aligned}$$

and $c_5:=8/(\pi c_v\mu _o^2).$ Now setting $\alpha =(\mu -\mu _o)\,(c_5+u)$ for sufficiently small and positive $\mu -\mu _o$ we get

$$\begin{aligned} u=\sum \limits _{n,m\ge 1} {\tilde{c}}_{n,m}(\mu - \mu _o)^n (c_5 + u)^m, \end{aligned}$$

where ${\tilde{c}}_{n,m}\subset {\mathbb {R}}.$ By the Implicit Function Theorem, for sufficiently small $\mu -\mu _o$ there exists a unique real-analytic function $u=u(\mu )$ given by the absolutely convergent series $u(\mu ) = \sum \limits _{n\ge 1} b_n(\mu - \mu _o)^n.$ By the definition of $\alpha ,$ this implies the expansion of $(-e(\mu ))^{1/4}.$

*Case* $2n_o+d=7.$ As the previous case, by (A.3) and (3.11), the equation (3.5) is represented as

3.12 $$\begin{aligned} (\mu - \mu _o)\left( c_7^3 + c_7^3\sum \limits _{n\ge 1} \mu _o^{-n}(\mu - \mu _o)^n\right) = \alpha ^{3} \left( 1 + \sum \limits _{n\ge 1}a_n\alpha ^n \right) , \end{aligned}$$

where $\{a_n\}\subset {\mathbb {R}}$ and $c_7:=[8/(\pi c_v\mu _o^2)]^{1/3}$. When $\mu -\mu _o>0$ is small enough, by the Taylor series of $(1+x)^{\pm 1/3}$ at $x=0,$ (3.12) is equivalently rewritten as

3.13 $$\begin{aligned} \alpha = (\mu - \mu _o)^{1/3}\left( c_7 + \sum \limits _{n\ge 1} {\tilde{c}}_n(\mu - \mu _o)^n\right) \left( 1 + \sum \limits _{n\ge 1}{\tilde{a}}_n\alpha ^n \right) , \end{aligned}$$

Thus, for $\rho =(\mu -\mu _o)^{1/3},$ setting $\alpha =\rho \,(c_7+u)$ in (3.13), for sufficiently small and positive $\rho$ we get

$$\begin{aligned} u=\sum \limits _{n,m\ge 1} {\tilde{c}}_{n,m}\rho ^n(c_7+u)^m. \end{aligned}$$

By the Implicit Function Theorem, this equation has a unique real-analytic solution $u=u(\rho )$ given by the absolutely convergent series $u=\sum \limits _{n\ge 1} b_n\rho ^n.$ This, definitions of $\alpha$ and $\rho$ imply the expansion of $(-e(\mu ))^{1/4}.$

*Case* $2n_o+d=9.$ In this case by (A.3) and (3.11)

3.14 $$\begin{aligned} (\mu - \mu _o)\left( c_9^4 + c_9^4\sum \limits _{n\ge 1} \mu _o^{-n}(\mu - \mu _o)^n\right) = \alpha ^4 \left( 1 + \sum \limits _{n\ge 1}a_n\alpha ^n \right) , \end{aligned}$$

where $\{a_n\}\subset {\mathbb {R}}$ and $c_9:=(\mu _o^2{\widehat{c}}_v)^{-1/4}.$ Thus, for sufficiently small and positive $\mu -\mu _o$ using the Taylor series of $(1+x)^{\pm 1/4}$ at $x=0,$ this equation can also be represented as

$$\begin{aligned} \alpha = \rho \left( c_9 + \sum \limits _{n\ge 1} {\tilde{b}}_n\rho ^{4n}\right) \left( 1 + \sum \limits _{n\ge 1}{\tilde{a}}_n\alpha ^n\right) , \end{aligned}$$

where $\rho := (\mu - \mu _o)^{1/4}.$ Now setting $\alpha = \rho (c_9+ u)$ in (3.14) we get

$$\begin{aligned} u=\sum \limits _{n,m\ge 1} {\tilde{c}}_{n,m}\rho ^n(c_9+u)^m, \end{aligned}$$

and the expansion of $(-e(\mu ))^{1/4}$ follows as in the case of $2n_o+d=7.$

(b) Suppose that *d* is even. In view of the expansion (A.3) of $\mathfrak {l}_f$, in this case, (3.4) is reduced to the inverting the equation

3.15 $$\begin{aligned} \mu = \frac{\alpha ^l}{g(\alpha ) + h(\alpha ) \ln \alpha }, \end{aligned}$$

where $\alpha :=(-e)^{1/2},$ $l\in {\mathbb {N}}_0,$ and *g* and *h* are analytic around $\alpha =0.$ Presence of $\ln \alpha$ implies that unlike the case of odd dimensions, $\alpha$ is not necessarily analytic with respect to $\mu ^s.$ Therefore, we need to introduce new variables dependent on $\ln \mu$ to reduce the problem to the Implicit Function Theorem.

*Case* $2n_o+d=2.$ By (A.4), in this case for $c_2:=\pi c_v/8$

$$\begin{aligned} l=1,\qquad g(\alpha )=c_2 + \sum \limits _{n\ge 1} a_n\alpha ^n,\qquad h(\alpha ) =\sum \limits _{n\ge 1} b_n\alpha ^{2n}. \end{aligned}$$

Hence, setting

3.16 $$\begin{aligned} \alpha =\mu (c_2 + u) \end{aligned}$$

and $\tau = -\mu \ln \mu$ we represent (3.15) as

$$\begin{aligned}&F(u,\mu ,\tau ):=u - \sum \limits _{n\ge 1} a^n\mu ^n(c_2+u)^n&+ \ln (c_2+u)\sum \limits _{n\ge 1} b^n\mu ^n(c_2+u)^n\\&\quad -\tau \sum \limits _{n\ge 1} b^n\mu ^{n-1}(c_2+u)^n=0, \end{aligned}$$

where *F* is analytic around (0, 0, 0),  $F(0,0,0)=0,$ $F_u(0,0,0)=1.$ Hence, by the Implicit Function Theorem, there exists a unique real-analytic function $u=u(\mu ,\tau )$ given by the convergent series $u(\mu ,\tau ) = \sum \nolimits _{n+m\ge 1,n,m\ge 0} {\tilde{c}}_{n,m}\mu ^n\tau ^m$ for sufficiently small $|\mu |$ and $|\tau |,$ which satisfies $F(u(\mu ,\tau ),\mu ,\tau )\equiv 0.$ Inserting *u* in (3.16) we get the expansion of $\alpha =(-e)^{1/2}.$

*Case* $2n_o+d=4.$ In this case, by (A.4) for $c_4:=8/ c_v$

$$\begin{aligned} l=0,\qquad g(\alpha )=\sum \limits _{n\ge 0} a_n\alpha _n,\qquad h(\alpha ) = -c_4 + \sum \limits _{n\ge 1} b_n\alpha ^{2n}. \end{aligned}$$

Letting $\alpha = e^{-\frac{1}{c_4\mu }}(c+u),$ where $c= e^{a_0/c_4}>0,$ we represent (3.15) as

3.17 $$\begin{aligned} \ln (c+u) - b_0 =&\frac{1}{\mu }\,e^{-\frac{1}{c_4\mu }} \sum \limits _{n\ge 1} a^ne^{-\frac{n-1}{c_4\mu }} (c+u)^n\nonumber \\ +&\ln (c+u)\sum \limits _{n\ge 1} b^ne^{-\frac{n}{c_4\mu }}(c+u)^n -\sum \limits _{n\ge 1} a^ne^{-\frac{n}{c_4\mu }}(c+u)^n=0. \end{aligned}$$

Writing $\tau :=\frac{1}{\mu }\,e^{-\frac{1}{c_4\mu }}$ so that $e^{-\frac{1}{c_4\mu }} = \mu \tau ,$ (3.17) is represented as

$$\begin{aligned} F(u,\mu ,\tau ):=&\ln (c+u) - b_0 -\mu \sum \limits _{n\ge 1} a^n\mu ^{n-1}\tau ^{n-1} (c+u)^n\\&-\ln (c+u)\sum \limits _{n\ge 1} b^n\mu ^n\tau ^n(c+u)^n +\sum \limits _{n\ge 1} a^n\mu ^n\tau ^n(c+u)^n=0, \end{aligned}$$

where *F* is analytic around (0, 0, 0),  $F(0,0,0)=0,$ and $F_u(0,0,0)=\frac{1}{c}>0.$ Thus, by the Implicit Function Theorem, for $|\mu |,$ $|\tau |$ and |*u*| small there exists a unique real analytic function $u=u(\mu ,\tau )$ given by the convergent series $u=\sum \limits _{n+m\ge 1,n,m\ge 0} {\tilde{c}}_{n,m}\mu ^n\tau ^m$ such that $F(u(\mu ,\tau ),\mu ,\tau )\equiv 0.$ Since $\tau = \frac{1}{\mu }e^{-\frac{1}{c_4\mu }},$ this implies

$$\begin{aligned} \alpha = e^{-\frac{1}{c_4\mu }}(c+u) = ce^{-\frac{1}{c_4\mu }} + \sum \limits _{n+m\ge 1,n,m\ge 0} {\tilde{c}}_{n,m}\mu ^{n+1}\left( \frac{1}{\mu }e^{-\frac{1}{c_4\mu }}\right) ^{m+1}. \end{aligned}$$

*Case* $2n_o+d=6.$ In this case, by (A.4), for $c_6:=8/(\pi c_v\mu _o^2)$

$$\begin{aligned} l=0,\qquad g(\alpha )=\frac{1}{\mu _o} -\frac{1}{c_6\mu _o^2} \left( \alpha + \sum \limits _{n\ge 2} a_n\alpha ^n\right) ,\qquad h(\alpha ) = \frac{1}{c_6\mu _o^2} \sum \limits _{n\ge 1} b_n\alpha ^{2n}, \end{aligned}$$

and hence, (3.15) is represented as

$$\begin{aligned} \frac{1}{\mu } -\frac{1}{\mu _o} = \frac{1}{c_6\mu _o^2}\left( \alpha +\sum \limits _{n\ge 2} a_n\alpha ^n + \ln \alpha \sum \limits _{n\ge 1} b_n\alpha ^{2n}\right) , \end{aligned}$$

or equivalently, by (3.11),

3.18 $$\begin{aligned} \alpha = c_6(\mu - \mu _o) \sum \limits _{n\ge 0} \left( \frac{\mu - \mu _o}{\mu _o}\right) ^n - \sum \limits _{n\ge 2} a_n\alpha ^n -\ln \alpha \sum \limits _{n\ge 1} b_n\alpha ^{2n}. \end{aligned}$$

Recalling the definitions of $\tau$ and $\theta$ in (2.8), setting $\alpha = \tau ^2\,(c_6+u),$ we represent (3.18) as

$$\begin{aligned} F(u,\tau , \theta ):= u&-c_6\sum \limits _{n\ge 1}\frac{\tau ^{2n}}{\mu _o^n}- \sum \limits _{n\ge 2} a_n\tau ^{2n-2}(c_6+u)^n\nonumber \\&- \ln (c_6+u) \sum \limits _{n\ge 1} b_n\tau ^{4n}(c_6+u)^{2n} - \theta \sum \limits _{n\ge 1} b_n\tau ^{4n-4}(c_6+u)^{2n}=0, \end{aligned}$$

where *F* is real-analytic around (0, 0, 0),  $F(0,0,0)=0$ and $F_u(0,0,0)=1,$ and *F* is even in $\tau .$ Thus, by the Implicit Function Theorem, for |*u*|,  $|\tau |$ and $|\theta |$ small there exists a unique real analytic function $u=u(\tau ,\theta ),$ even in $\tau ,$ given by the convergent series $u=\sum \limits _{n+m\ge 1,n,m\ge 0} {\tilde{c}}_{n,m}\tau ^{2n}\theta ^m$ such that $F(u(\tau ,\theta ),\tau ,\theta )\equiv 0.$ Thus,

$$\begin{aligned} \alpha = \tau ^2\,(c_6+u) = c_6\sigma + \sum \limits _{n+m\ge 1,n,m\ge 0} {\tilde{c}}_{n,m}\tau ^{2n+2}\theta ^m. \end{aligned}$$

*Case* $2n_o+d=8.$ By (A.4), for $c_8:=[8/c_v\mu _o^2]^{-1/2},$

$$\begin{aligned} l=0,\qquad g(\alpha )= \frac{1}{\mu _o^2c_8^2}\sum \limits _{n\ge 2} a_n\alpha ^n,\qquad h(\alpha ) =\frac{1}{\mu _o^2c_8^2}\left( \alpha ^2 + \sum \limits _{n\ge 2} b_n\alpha ^{2n}\right) , \end{aligned}$$

thus, as in the case of $2n_o+d=6,$ (3.15) is represented as

3.19 $$\begin{aligned} c_8^2(\mu - \mu _o) \sum \limits _{n\ge 0} \left( \frac{\mu - \mu _o}{\mu _o}\right) ^n = \alpha ^2\ln \alpha +\ln \alpha \sum \limits _{n\ge 2} b_n\alpha ^{2n}+\sum \limits _{n\ge 2} a_n\alpha ^n. \end{aligned}$$

For $\tau ,$ $\sigma$ and $\eta$ given in (2.8) set $\alpha = \tau \sigma (c_8+u)$ and represent (3.19) as

$$\begin{aligned} 2c_8u+ u^2 =&c_8^2\sum \limits _{n\ge 1} \frac{\tau ^{2n}}{\mu _o^n} + \sum \limits _{n\ge 2}a_n \tau ^{n-1} \sigma ^{n+1}(c_8+u)^{n+2} \\&-\sum \limits _{n\ge 2}b_n (\tau \sigma )^{2n-2}(c_8+u)^{2n+2}\\&+ \left( \sigma ^2\ln (c_8+u) -\frac{\eta }{2} \right) \left( (c_8+u)^2+\sum \limits _{n\ge 2}b_n (\tau \sigma )^{2n-2}(c_8+u)^{2n+2}\right) . \end{aligned}$$

This equation is represented as $F(u,\tau ,\sigma ,\eta )=0,$ where *F* is real-analytic in a neighborhood of (0, 0, 0, 0),  $F(0,0,0,0)=0$ and $F_u(0,0,0,0) =2c_8>0.$ Hence, for |*u*|,  $|\tau |,$ $|\sigma |$ and $|\eta |$ small, by the Implicit Function Theorem, there exists a unique real-analytic function $u=u(\tau ,\sigma ,\eta )$ given by the convergent series $u=\sum \limits _{n+m+k\ge 1,n,m,k\ge 0} {\tilde{c}}_{n,m,k}\tau ^n\sigma ^m\mu ^k$ such that $F(u(\tau ,\sigma ,\eta ),\tau ,\sigma ,\eta )\equiv 0.$ Thus,

$$\begin{aligned} \alpha = \tau \sigma (c_8+u) =c_8\tau \sigma + \sum \limits _{n+m+k\ge 1,n,m,k\ge 0} {\tilde{c}}_{n,m,k}\tau ^{n+1}\sigma ^{m+1}\eta ^k. \end{aligned}$$

*Case* $2n_o+d\ge 10.$ By (A.4) for $c_{10}:=(\mu _o^2{\widehat{c}}_v)^{-1/2},$

$$\begin{aligned} l=0,\qquad g(\alpha )= \frac{1}{\mu _o} + {\widehat{c}}_v \alpha ^2 + \sum \limits _{n\ge 2} a_n\alpha ^{n+2},\qquad h(\alpha ) =\sum \limits _{n\ge 2} b_n\alpha ^{2n}, \end{aligned}$$

and as in the case of $2n_o+d=6,$ (3.15) is represented as

3.20 $$\begin{aligned} \frac{\mu - \mu _o}{\mu _o^2} \sum \limits _{n\ge 0} \left( \frac{\mu - \mu _o}{\mu _o}\right) ^n = {\widehat{c}}_v \alpha ^2 + \sum \limits _{n\ge 2} a_n\alpha ^{n+2}+ \ln \alpha \sum \limits _{n\ge 2} b_n\alpha ^{2n}. \end{aligned}$$

Recalling the definitions of $\tau$ and $\theta$ in (2.8), we set $\alpha =\tau (c_{10} +u).$ Then (3.20) is represented as

$$\begin{aligned} F(u,\tau ,\theta ):=&2c_{10}u + u^2 - c_{10}^2\sum \limits _{n\ge 1} \frac{\tau ^{2n}}{\mu _o^n} + \sum \limits _{n\ge 2}a_n \tau ^n (c_{10}+u)^{n+2}\\&- \theta \sum \limits _{n\ge 2}b_n \tau ^{2n-4}(c_8+u)^{2n} + \ln (c_{10}+u)\sum \limits _{n\ge 2}b_n \tau ^{2n-2}(c_8+u)^{2n}=0, \end{aligned}$$

where *F* is analytic at (0, 0, 0),  $F(0,0,0)=0$ and $F_u(0,0,0)=2c_{10}>0.$ Thus, by the Implicit Function Theorem, for |*u*|,  $|\tau |$ and $|\theta |$ small there exists a unique real-analytic function $u=u(\tau ,\theta )$ given by the convergent series $u=\sum \limits _{n+m\ge 1,n,m\ge 0}{\tilde{c}}_{n,m}\tau ^n\theta ^n$ such that $F(u(\tau ,\theta ),\tau ,\theta )\equiv 0.$ Then

$$\begin{aligned} \alpha = \mu (c_{10} +u) = c_{10}\mu + \sum \limits _{n+m\ge 1,n,m\ge 0}{\tilde{c}}_{n,m}\mu ^{n+1}\theta ^n. \end{aligned}$$

Theorem is proved. $\square$

### Proof of Theorem 2.5

From (3.3) it follows that the map $p\in {\mathbb {T}}^d\mapsto |v|^2(\vec \pi +p)$ is even. Now the expansions of $e(\mu )$ at $\mu =-\mu ^o$ can be proven along the same lines of Theorem 2.4 using Proposition A.2 with $f=|v|^2$. $\square$

### Remark 3.3

Let ${\widehat{v}}:{\mathbb {Z}}^d\rightarrow {\mathbb {C}}$ satisfy (H1). Since ${\mathfrak {e}}(\cdot )$ is even,

$$\begin{aligned} \int _{{\mathbb {T}}^d} \frac{|v(p)|^2dp}{{\mathfrak {e}}(p) -z} =\frac{1}{(2\pi )^d} \, \int _{{\mathbb {T}}^d} \frac{f(p)dp}{{\mathfrak {e}}(p) -z}, \end{aligned}$$

where

$$\begin{aligned} \begin{aligned} f(p):=&\left( \sum \limits _{x\in {\mathbb {Z}}^d} {\widehat{v}}_1(x) \cos p\cdot x\right) ^2 + \left( \sum \limits _{x\in {\mathbb {Z}}^d} {\widehat{v}}_2(x) \cos p\cdot x\right) ^2\\&+ \left( \sum \limits _{x\in {\mathbb {Z}}^d} {\widehat{v}}_1(x) \sin p\cdot x\right) ^2 + \left( \sum \limits _{x\in {\mathbb {Z}}^d} {\widehat{v}}_2(x) \sin p\cdot x\right) ^2 \end{aligned} \end{aligned}$$

and ${\widehat{v}}= {\widehat{v}}_1+ i{\widehat{v}}_2$ for some ${\widehat{v}}_1,{\widehat{v}}_2:{\mathbb {Z}}^d \rightarrow {\mathbb {R}}.$ By Lemma 3.1, the unique eigenvalue $e(\mu )$ of ${ H}_\mu$ solves

$$\begin{aligned} 1-\mu \int _{{\mathbb {T}}^d} \frac{f(p)dp}{{\mathfrak {e}}(p) - e(\mu )} =0. \end{aligned}$$

Since both $p\in {\mathbb {T}}^d\mapsto f(p)$ and $p\in {\mathbb {T}}^d\mapsto f(\vec \pi + p)$ are even analytic functions, we can still apply Propositions A.1 and A.2 to find the expansions of $z\mapsto \int _{{\mathbb {T}}^d} \frac{f(p)dp}{{\mathfrak {e}}(p) - z}$ and thus, repeating the same arguments of the proofs of Theorems 2.4 and 2.5 one can obtain the corresponding expansions of $e(\mu ).$

### Remark 3.4

When

$$\begin{aligned} |{\widehat{v}}(x)| = O(|x|^{2n_0+d+1})\qquad \hbox { as}\ |x|\rightarrow \infty \end{aligned}$$

for some $n_0\ge 1,$ in view of Remark A.3, we need to solve equation (3.4) with respect to $\mu$ using only that left-hand side is an asymptotic sum (not a convergent series). This still can be done using appropriate modification of the Implicit Function Theorem for differentiable functions. As a result, we obtain only (Taylor-type) asymptotics of $e(\mu ).$

## Appendix A. Asymptotics of some integrals

In this section we study the behaviour of the integral

A.1 $$\begin{aligned} \mathfrak {l}_f(z):=\int _{{\mathbb {T}}^d} \frac{f(q)dq}{{\mathfrak {e}}(q) -z},\qquad z\in {\mathbb {C}}\setminus [0,4d^2], \end{aligned}$$

as $z\rightarrow 0$ and $z\rightarrow 4d^2,$ where $f:{\mathbb {T}}^d\rightarrow {\mathbb {R}}$ is a real-analytic even function on ${\mathbb {T}}^d.$ Further we denote by $W_r(\xi )\subset {\mathbb {C}}$ the complex disc of radius $r>0$ centered at $\xi \in {\mathbb {C}}.$

### Proposition A.1

Let $f:{\mathbb {T}}^d\rightarrow {\mathbb {R}}$ be a real-analytic even function such that

A.2 $$\begin{aligned} f(0) = D^2f(0) = \ldots =D^{2n_o-2}f(0) = 0,\qquad D^{2n_o}(0)\ne 0 \end{aligned}$$

for some $n_o\ge 0.$ Then:

- $\mathfrak {l}_f$ is continuous at 0 if and only if $2n_o+d\ge 5;$
- $\mathfrak {l}_f$ is continuously differentiable at 0 if and only if $2n+d\ge 9,$ in this case,
  $$\begin{aligned} \mathfrak {l}_f'(0):=\int _{{\mathbb {T}}^d} \frac{f(q) dq}{({\mathfrak {e}}(q))^2}= \lim \limits _{z\searrow 0} \int _{{\mathbb {T}}^d} \frac{f(q)dq}{({\mathfrak {e}}(q) -z)^2}. \end{aligned}$$

Moreover, for any $z\in (-\frac{1}{64},0):$

- if *d* is odd, then
  A.3 $$\begin{aligned} \mathfrak {l}_f(z) = {\left\{ \begin{array}{ll} \frac{\pi }{4(-z)^{3/4}}\, \left( c_f+ \sum \limits _{n\ge 1} a_n^d\,(-z)^{n/4}\right) , &{} 2n_o+d=1,\\ \frac{\pi }{8(-z)^{1/4}}\, \left( c_f+ \sum \limits _{n\ge 1} a_n^d\,(-z)^{n/4}\right) , &{} 2n_o+d=3,\\ \mathfrak {l}_f(0)-\frac{\pi (-z)^{1/4}}{8}\,\left( c_f+ \sum \limits _{n\ge 1} a_n^d\,(-z)^{n/4}\right) , &{} 2n_o+d=5,\\ \mathfrak {l}_f(0)-\frac{\pi (-z)^{3/4}}{8}\,\left( c_f+ \sum \limits _{n\ge 1} a_n^d\,(-z)^{n/4}\right) , &{} 2n_o+d=7,\\ \mathfrak {l}_f(0) + z\left( \mathfrak {l}_f'(0) + \sum \limits _{n\ge 1} a_n^d\,(-z)^{n/4}\right) , &{} 2n_o+d\ge 9, \end{array}\right. } \end{aligned}$$
- if *d* is even, then
  A.4 $$\begin{aligned} \mathfrak {l}_f(z) = {\left\{ \begin{array}{ll} \frac{\pi }{8(-z)^{1/2}}\, \left( c_f+ \sum \limits _{n\ge 1} b_n^d\,(-z)^{n/2}\right) -\frac{1}{16}\,\ln (-z)\sum \limits _{n\ge 0} c_n^dz^n , &{} 2n_o+d=2,\\ -\frac{1}{16}\,\ln (-z)\left( c_f + \sum \limits _{n\ge 1} c_n^dz^n\right) +\sum \limits _{n\ge 0} b_n^d\,(-z)^{n/2},&{} 2n_o+d=4,\\ \mathfrak {l}_f(0)-\frac{\pi (-z)^{1/2}}{8}\, \left( c_f+ \sum \limits _{n\ge 1} b_n^d\,(-z)^{n/2}\right) +z\ln (-z)\sum \limits _{n\ge 0} c_n^dz^n , &{} 2n_o+d=6,\\ \mathfrak {l}_f(0)- \frac{z}{16}\,\ln (-z)\left( c_f + \sum \limits _{n\ge 1} c_n^dz^n\right) +\sum \limits _{n\ge 2} b_n^d\,(-z)^{n/2},&{} 2n_o+d=8,\\ \mathfrak {l}_f(0) + z\left( \mathfrak {l}_f'(0) + \sum \limits _{n\ge 1} b_n^d\,(-z)^{n/2}\right) + z^2\ln (-z)\sum \limits _{n\ge 0} c_n^d\,z^n , &{} 2n_o+d\ge 10, \end{array}\right. }\nonumber \\ \end{aligned}$$

where $\{a_n^d\},$ $\{b_n^d\}$ and $\{c_n^d\}$ are some real coefficients,

A.5 $$\begin{aligned} c_f:=\frac{2^{2n_o+d}}{(2n_o)!}\, \int _{{\mathbb {S}}^{d-1}} D^{2n_o}f(0)[w,\ldots ,w]d{\mathcal {H}}^{d-1}; \end{aligned}$$

and all series in (A.3) and (A.4) converge absolutely for $z\in W_{1/64}(0)\subset {\mathbb {C}}.$

### Proof

Given $\gamma \in (0,\frac{1}{\sqrt{2}}],$ let $\varphi :B_{\gamma }(0)\subset {\mathbb {R}}^d\rightarrow \varphi (B_{\gamma }(0))\subset {\mathbb {R}}^d$ be the smooth diffeomorphism

$$\begin{aligned} \varphi _i(y) = 2 \arcsin y_i,\qquad i=1,\ldots ,d. \end{aligned}$$

Note that

A.6 $$\begin{aligned} {\mathfrak {e}}(\varphi (y)) = \left( \sum \limits _{i=1}^d (1 - \cos (2\arcsin (y_i)) )\right) ^2 = 4\left( \sum \limits _{i=1}^d y_i^2\right) ^2 =4 y^4, \end{aligned}$$

therefore,

A.7 $$\begin{aligned} {\mathfrak {e}}(q)\ge 4 \gamma ^4 \qquad \hbox { for any}\ q\in {\mathbb {T}}^d\setminus \varphi (B_{\gamma }). \end{aligned}$$

We rewrite $\mathfrak {l}_f(z)$ as

$$\begin{aligned} \mathfrak {l}_f(z): = \int _{\varphi (B_{\gamma }(0))} \frac{f(q)dq}{{\mathfrak {e}}(q) -z} + \int _{{\mathbb {T}}^d\setminus \varphi (B_{\gamma }(0))} \frac{f(q)dq}{{\mathfrak {e}}(q) -z}:=\mathfrak {l}^*(z) +\mathfrak {l}^{**}(z). \end{aligned}$$

By virtue of (A.7),

A.8 $$\begin{aligned} \mathfrak {l}^{**}(z) = \int _{{\mathbb {T}}^d\setminus \varphi (B_{\gamma }(0))} \frac{f(q)}{{\mathfrak {e}}(q)}\,\left( 1-\frac{z}{{\mathfrak {e}}(q)}\right) ^{-1}dq =\sum \limits _{n\ge 0} z^{n} \int _{{\mathbb {T}}^d\setminus \varphi (B_{\gamma }(0))} \frac{f(q)dq}{({\mathfrak {e}}(q))^{n+1}},\nonumber \\ \end{aligned}$$

i.e. $$\mathfrak {l}^{**}(\cdot )$$ is analytic in $$W_{2\gamma ^4}(0).$$ In $$\mathfrak {l}^*$$ making the change of variables $$q = \varphi (y)$$ and using (A.6) we get

A.9 $$\begin{aligned} \mathfrak {l}^*(z) = \int _{B_{\gamma }(0)} \frac{f(\varphi (y))\, J(\varphi (y))\,dy}{4y^4 -z}, \end{aligned}$$

where $y^4:=(y^2)^2$ with $y^2:=\sum \nolimits _{i=1}^d y_i^2$, and

A.10 $$\begin{aligned} J(\varphi (y)) = \prod \limits _{i=1}^d \frac{2}{\sqrt{1-y_i^2}} \end{aligned}$$

is the Jacobian of $\varphi .$ Since *f* is an even analytic function satisfying (A.2), even each coordinate, from the Taylor series for *f* it follows that

A.11 $$\begin{aligned} f(p) = \sum \limits _{n\ge n_o} \frac{1}{(2n)!}\,D^{2n}f(0)[\underbrace{p,\ldots ,p}_{\text {2{ n}-times}}], \end{aligned}$$

and by the analyticity of *f* in $B_\pi (0)\subset {\mathbb {R}}^d,$ the series converges absolutely in $p\in B_\pi (0).$ By the definition of $\varphi ,$ $\varphi (rw)\subset B_\pi (0)$ for any $r\in (0,\gamma )$ and $w=(w_1,\ldots ,w_d)\in {\mathbb {S}}^{d-1},$ where ${\mathbb {S}}^{d-1}$ is the unit sphere in ${\mathbb {R}}^d.$ Then letting $p=\varphi (rw)$ and using the Taylor series

$$\begin{aligned} \varphi _i(rw)= 2rw_i + \frac{r^3w_i^3}{3} +\sum \limits _{n\ge 3} {\tilde{c}}_n r^{2n-1}w_i^{2n-1} \end{aligned}$$

of $2\arcsin (\cdot ),$ which is absolutely convergent for $|rw_i|<1,$ from (A.11) we obtain

A.12 $$\begin{aligned} f(\varphi (rw))= \sum \limits _{n\ge n_o} {\tilde{C}}_n(w)\,r^{2n}, \end{aligned}$$

where ${\tilde{C}}_n:{\mathbb {S}}^{d-1}\rightarrow {\mathbb {R}}$ is a homogeneous polynomial of $w\in {\mathbb {S}}^{d-1}$ of degree 2*n*,  and

$$\begin{aligned} {\tilde{C}}_{n_o}(w) = \frac{{2^{{2n_{o} }} }}{{\left( {2n_{o} } \right) !}}{} D^{{2n_{o} }} f(0)\left[ {\underbrace{{w, \ldots ,w}}_{{{\text {2n}}_{{\text {o}}} {\text { - times}}}}} \right] \end{aligned}$$

Next consider $J(\varphi (y)).$ Inserting the Taylor series of $(1-t)^{-1/2}$ into (A.10) we obtain

A.13 $$\begin{aligned} J(\varphi (rw)) = 2^d\left( 1 + \sum \limits _{n\ge 1} {\widehat{C}}_n(w)r^{2n}\right) , \end{aligned}$$

where ${\widehat{C}}_n:{\mathbb {S}}^{d-1}\rightarrow {\mathbb {R}}$ is a homogeneous symmetric polynomial of $w\in {\mathbb {S}}^{d-1}$ of degree 2*n*,  and the series converges absolutely.

Now passing to polar coordinates by $y=rw$ in (A.9) and using (A.12) and (A.13) as well as the absolute convergence of the series we get

A.14 $$\begin{aligned} \mathfrak {l}^*(z) =&2^d\int _0^\gamma \frac{r^{d-1}}{4r^4-z}\, \left( \sum \limits _{n\ge n_o} \int _{{\mathbb {S}}^{d-1}} C_n(w)\,r^{2n}\right) d{\mathcal {H}}^{d-1}dr = \sum \limits _{n\ge n_o} {\widehat{c}}_n \int _0^\gamma \frac{r^{2n+d-1}dr}{4r^4-z}, \end{aligned}$$

where $C_n:{\mathbb {S}}^{d-1}\rightarrow {\mathbb {R}}$ is a homogeneous polynomial of $w\in {\mathbb {S}}^{d-1}$ of degree 2*n* and

$$\begin{aligned} {\widehat{c}}_n:=2^d\int _{{\mathbb {S}}^{d-1}} C_n(w)d{\mathcal {H}}^{d-1}. \end{aligned}$$

Note that ${\widehat{c}}_{n_o}=c_f,$ where $c_f$ is given by (A.5) and the last series in (A.14) uniformly converges in any compact subset of ${\mathbb {C}}\setminus [0,4]$ since $\mathfrak {l}^*$ and

$$\begin{aligned} z\in {\mathbb {C}}\setminus [0,4]\mapsto \mathfrak {j}_{2n+d-1}(z):=\int _0^\gamma \frac{r^{2n+d-1}dr}{4r^4-z} \end{aligned}$$

are analytic functions in ${\mathbb {C}}\setminus [0,4]$ and all series in (A.14) converge pointwise^1^. Note that for any $m\ge 0,$ there exist $c_m\in {\mathbb {R}}$ and an analytic function $f_m$ in the ball $W_{\gamma ^4}(0)\subset {\mathbb {C}}$ such that for any $z\in (-\gamma ^4,0),$

A.15 $$\begin{aligned} \mathfrak {j}_m(z) = z^n\,\, \mathfrak {j}_l^o(z) + c_m+ z^\nu f_m((-z)^{1/2}), \end{aligned}$$

where $n:=[\frac{m}{4}],$ $l:=m-4n\in \{0,1,2,3\},$ $\nu =\frac{1}{2}$ for $m=0,2$ and $\nu =1$ for $m=1,3$ or $m\ge 4,$ and

$$\begin{aligned} \mathfrak {j}_l^o (z):= {\left\{ \begin{array}{ll} \frac{\pi }{4}\,(-z)^{-3/4} &{}\qquad \hbox { if}\ \quad l=0,\\ \frac{\pi }{8}\,(-z)^{-1/2} &{}\qquad \hbox { if}\ \quad l=1,\\ \frac{\pi }{8}\,(-z)^{-1/4} &{} \qquad \hbox { if}\ \quad l=2,\\ -\frac{1}{16}\,\ln (-z) &{} \qquad \hbox { if}\ \quad l=3. \end{array}\right. } \end{aligned}$$

Inserting (A.15) into (A.14) we obtain

$$\begin{aligned} \mathfrak {l}^*(z) =&\sum \limits _{n\ge n_o} {\widehat{c}}_n\left( z^{[\frac{2n+d-1}{4}]}\, \mathfrak {j}_{2n+d-1 - 4[\frac{2n+d-1}{4}]}^o (z)\right. \\&\qquad \left. + c_{2n+d-1} +{\widehat{c}}_n (-z)^{\nu _n}f_{2n+d-1}((-z)^{1/2})\right) , \end{aligned}$$

where $\{c_{2n+d-1}\}\subset {\mathbb {R}}$ and $\{f_{2n+d-1}\}$ is a sequence of analytic functions in $W_{\gamma ^4}(0)$ and

$$\begin{aligned} \nu _n:= {\left\{ \begin{array}{ll} \frac{1}{2}, &{} 2n+d=1,3,\\ 1, &{} \text {otherwise}. \end{array}\right. } \end{aligned}$$

Since (A.14) converges locally uniformly in ${\mathbb {C}}\setminus [0,4],$ $C:=\sum \limits _{n\ge n_o} {\widehat{c}}_n c_{2n+d-1}$ is finite and

$$\begin{aligned} \sum \limits _{n\ge n_o} {\widehat{c}}_n (-z)^{\nu _n} f_{2n+d-1}((-z)^{1/2})= (-z)^\nu g((-z)^{1/2}), \end{aligned}$$

where *g* is analytic in $W_{\gamma ^2}(0)$ and $\nu =\frac{1}{2}$ for $2n_o+d=1,3$ and $\nu =1$ otherwise. Hence,

A.16 $$\begin{aligned} \mathfrak {l}^*(z) =C+ (-z)^\nu g((-z)^{1/2}) + \sum \limits _{n\ge n_o} {\widehat{c}}_n\,z^{[\frac{2n+d-1}{4}]}\, \mathfrak {j}_{2n+d-1 - 4[\frac{2n+d-1}{4}]}^o (z), \end{aligned}$$

If $0\le 2n_o+d-1\le 3,$ then by (A.16),

A.17 $$\begin{aligned} \mathfrak {l}^*(z)&= C+ (-z)^{\nu }g((-z)^{1/2}) + {\widehat{c}}_{n_o} \mathfrak {j}_{2n_o+d-1}^o(z) \nonumber \\&+ \sum \limits _{n\ge n_o+1} {\widehat{c}}_n z^{[\frac{2n+d-1}{4}]}\, \mathfrak {j}_{2n+d-1 - 4[\frac{2n+d-1}{4}]}^o(z). \end{aligned}$$

In view of (A.8) and the definition of $\mathfrak {j}_l^o,$ from (A.17) we obtain the expansions (A.3) and (A.4) of $\mathfrak {l}_f$ for $2n_o+d\le 4.$ In particular, since $[\frac{2n+d-1}{4}]\ge 1$ for $n\ge n_o+1,$ letting $z\rightarrow 0$ in (A.17) we get

A.18 $$\begin{aligned} \lim \limits _{z\rightarrow 0} \mathfrak {l}^*(z) =+\infty . \end{aligned}$$

If $2n_o+d-1\ge 4,$ then $[\frac{2n+d-1}{4}]\ge 1$ for any $n\ge n_o.$ Therefore, by (A.16), $\mathfrak {l}^*(0):=\lim \limits _{z\rightarrow 0} \mathfrak {l}^*(z)$ exists and equals to *C*. In particular, for $2n_o+d-1\le 7,$ one has

A.19 $$\begin{aligned} \mathfrak {l}^*(z)&= \mathfrak {l}^*(0) -z g((-z)^{1/2}) + {\widehat{c}}_{n_o} z \mathfrak {j}_{2n_o+d-1}^o(z)\nonumber \\&+ \sum \limits _{n\ge n_o+1} {\widehat{c}}_n z^{[\frac{2n+d-1}{4}]}\, \mathfrak {j}_{2n+d-1 - 4[\frac{2n+d-1}{4}]}^o(z), \end{aligned}$$

from which and (A.8) we deduce the expansions (A.3) and (A.4) of $$\mathfrak {l}_f$$ for $$5\le 2n_o+d\le 8.$$ In particular, by virtue of (A.18) and analyticity of $$\mathfrak {l}^{**}$$ at $$z=0,$$ $$\mathfrak {l}_f$$ is continous at 0 if and only if $$2n_o+d\ge 5.$$ Notice also by (A.19)

A.20 $$\begin{aligned} \lim \limits _{z\rightarrow 0 } \frac{\mathfrak {l}^*(z) - \mathfrak {l}^*(0)}{z} =+\infty , \end{aligned}$$

i.e. $\mathfrak {l}^*$ (and hence $\mathfrak {l}_f$) is not differentiable at $z=0.$

Finally, if $2n_o+d-1\ge 8,$ then $[\frac{2n+d-1}{4}]\ge 2$ for any $n\ge n_o.$ Therefore, by (A.16) there exists

$$\begin{aligned} {\mathfrak {l}^*}'(0):=\lim \limits _{z\rightarrow 0 } \frac{\mathfrak {l}^*(z) - \mathfrak {l}^*(0)}{z} =- g(0). \end{aligned}$$

Now using the Taylor series of *g* at 0 we get

$$\begin{aligned} zg((-z)^{1/2})= {\mathfrak {l}^*}'(0) z + z\sum \limits _{n\ge 1}\frac{g^{(n)}(0)}{n!}\, (-z)^{n/2}. \end{aligned}$$

Inserting this in (A.16), using the definition of $$\mathfrak {j}_l^o$$ and the analyticity of $$\mathfrak {l}^{**}$$ we get the expansions (A.3) and (A.4) of $$\mathfrak {l}_f$$ for $$2n_o+d\ge 9.$$

By (A.18) and (A.20), $\mathfrak {l}_f$ is continously differentiable at 0 if and only if $2n_o+d\ge 9.$

Now the choice $\gamma =\frac{1}{\sqrt{2}}$ completes the proof. $\square$

### Proposition A.2

Let $f:{\mathbb {T}}^d\rightarrow {\mathbb {R}}$ be a real-analytic function such that $q\in {\mathbb {T}}^d\mapsto f(\vec \pi +q)$ is even and

$$\begin{aligned} f(\vec \pi ) = D^2f(\vec \pi ) = \ldots =D^{2n_o-2}f(\vec \pi ) = 0,\qquad D^{2n_o}(\vec \pi )\ne 0 \end{aligned}$$

for some $n_o\in {\mathbb {N}}_0.$ Then:

- $\mathfrak {l}_f$ is continuous at $z=4d^2$ if and only if for $2n_o+d\ge 3,$
- $\mathfrak {l}_f$ is continuously differentiable at $z=4d^2$ if and only if for $2n_o+d\ge 5,$ in this case
  $$\begin{aligned} \mathfrak {l}_f'(4d^2):=\int _{{\mathbb {T}}^d} \frac{f(q) dq}{({\mathfrak {e}}(q)-4d^2)^2}= \lim \limits _{z\searrow 4d^2} \int _{{\mathbb {T}}^d} \frac{f(q)dq}{({\mathfrak {e}}(q) - z)^2} \end{aligned}$$
  exists.

Moreover, if $z-4d^2\in (0,\frac{1}{16}),$ $\mathfrak {l}_f(z)$ is represented as:

- if *d* is odd, then
  A.21 $$\begin{aligned} \mathfrak {l}_f(z) = {\left\{ \begin{array}{ll} -\frac{\pi C_f}{\sqrt{z-4d^2}} + \sum \limits _{k\ge 0} a_k^d(z-4d^2)^{k/2}, &{} 2n_o+d=1,\\ \mathfrak {l}_f(4d^2) + \pi C_f\,\sqrt{z-4d^2} + \sum \limits _{k\ge 2} a_k^d(z-4d^2)^{k/2}, &{} 2n_o+d=3,\\ \mathfrak {l}_f(4d^2) + \mathfrak {l}_f'(4d^2)\,(z-4d^2) + \sum \limits _{k\ge 3} a_k^d(z-4d^2)^{k/2}, &{} 2n_o+d\ge 5; \end{array}\right. }\nonumber \\ \end{aligned}$$
- if *d* is even, then
  A.22 $$\begin{aligned} \mathfrak {l}_f(z) = {\left\{ \begin{array}{ll} C_f \ln \alpha + \ln \alpha \sum \limits _{k\ge 1} b_k^d \alpha ^k + \sum \limits _{k\ge 0} c_k^d\alpha ^k, &{} 2n_o+d=2,\\ \mathfrak {l}_f(4d^2) - C_f\alpha \ln \alpha + \ln \alpha \sum \limits _{k\ge 2} b_k^d \alpha ^k + \sum \limits _{k\ge 1} c_k^d\alpha ^k, &{} 2n_o+d=4,\\ \mathfrak {l}_f(4d^2) + \mathfrak {l}_f'(4d^2)\,\alpha + \ln \alpha \sum \limits _{k\ge 2} b_k^d \alpha ^k + \sum \limits _{k\ge 2} c_k^d\alpha ^k, &{} 2n_o+d\ge 6, \end{array}\right. }\nonumber \\ \end{aligned}$$

where $$\alpha :=z-4d^2,$$ $$\{a_k^d\},\{b_k^d\},\{c_k^d\}\subset {\mathbb {R}}$$ and

$$\begin{aligned} C_f:= \frac{2^{2n_o+d-1}}{(8d)^{n_o+d/2}\,(2n_o)!}\,\int _{{\mathbb {S}}^{d-1}} D^{2n_o}f(\vec \pi )[w,\ldots ,w]d{\mathcal {H}}^{d-1}. \end{aligned}$$

### Proof

Since $4d^2-{\mathfrak {e}}(\cdot )$ has a unique non-degenerate minimum at $\vec \pi ,$ the asymptotics of $\mathfrak {l}_f(z)$ as $z\searrow 4d^2$ can be done along the lines of, for instance, [22, Lemma 4.1], hence, we skip the proof. $\square$

### Remark A.3

When

$$\begin{aligned} |{\widehat{v}}(x)| = O(|x|^{2n_0+d+1})\qquad \hbox { as}\ |x|\rightarrow \infty \end{aligned}$$

for some $n_0\ge 1,$ one has $v\in C^{2n_0}({\mathbb {T}}^d).$ In this case the Taylor series of *f* becomes only asymptotics of order $2n_0-1$ and thus, instead of expansions (A.3)-(A.4) and (A.21)-(A.22) of $\mathfrak {l}_f$ one has only asymptotics up to order $2n_0-1.$
